# Capturing coevolutionary signals inrepeat proteins

**DOI:** 10.1186/s12859-015-0648-3

**Published:** 2015-07-02

**Authors:** Rocío Espada, R Gonzalo Parra, Thierry Mora, Aleksandra M Walczak, Diego U Ferreiro

**Affiliations:** 1 Protein Physiology Lab, Dep de Química Biológica, Facultad de Ciencias Exactas y Naturales, UBA-CONICET-IQUIBICEN, Buenos Aires, Argentina; 2 Laboratoire de physique statistique, CNRS, UPMC and École normale supérieure, 24 rue Lhomond, Paris, 75005 France; 3 24 rue Lhomond, Paris, 75005 France; 4 Departamento de Física, Facultad de Ciencias Exactas y Naturales, Universidad de Buenos Aires, Buenos Aires, Argentina

**Keywords:** Direct coupling analysis, Repeat proteins, Direct information, Co-evolution

## Abstract

**Background:**

The analysis of correlations of amino acid occurrences in globular domains has led to the development of statistical tools that can identify native contacts – portions of the chains that come to close distance in folded structural ensembles. Here we introduce a direct coupling analysis for repeat proteins – natural systems for which the identification of folding domains remains challenging.

**Results:**

We show that the inherent translational symmetry of repeat protein sequences introduces a strong bias in the pair correlations at precisely the length scale of the repeat-unit. Equalizing for this bias in an objective way reveals true co-evolutionary signals from which local native contacts can be identified. Importantly, parameter values obtained for all other interactions are not significantly affected by the equalization. We quantify the robustness of the procedure and assign confidence levels to the interactions, identifying the minimum number of sequences needed to extract evolutionary information in several repeat protein families.

**Conclusions:**

The overall procedure can be used to reconstruct the interactions at distances larger than repeat-pairs, identifying the characteristics of the strongest couplings in each family, and can be applied to any system that appears translationally symmetric.

**Electronic supplementary material:**

The online version of this article (doi:10.1186/s12859-015-0648-3) contains supplementary material, which is available to authorized users.

## Background

The fact that many protein molecules spontaneously collapse stretches of amino acid chains into defined structural domains [1] facilitates the description, evolution and construction of these peculiar physical objects. Higher order biological *functions* that correlate with domains can usually be isolated, recombined and adjusted, akin to engineering [2], or tinkering [3] using modular components. The evolutionary record of natural proteins results from a balance between sequence exploration and constraints: conservation of function within a protein family imposes strong boundaries on sequence variation, sculpting the structural forms visited by members of a protein family. Amino acids that are in spatial proximity in the mean conformational ensemble are expected to co-vary on evolutionary timescales, as the energy contributions to fold stabilization can be often localized to groups of residues [4]. However, correlated residue changes throughout proteins’ history may not necessarily be close in space, as other constraints are always at play [5]. Since the evolutionary record is inevitably incomplete, the sequences we find today constitute a biased sample of the possible outcomes, therefore any search for the underlying constraints must take into account contingent factors that may confound the observed correlations. Here we use sequence correlations to explore the link between structure and function in repeat proteins, natural systems for which the identification of functional domains remains challenging [6].

Many natural proteins contain tandem repeats of similar amino acid stretches. Repeat proteins represent close to 6 % of polipeptide sequences codified in eukaryotic genomes [7]. These have been broadly classified in groups according to the length of the minimal repeating units [8]. Still, there are open problems of quantitatively defining the repeat protein families, the number and location of the repeat occurrences and the grouping of these into repeat-arrays.

**Fig. 1:**
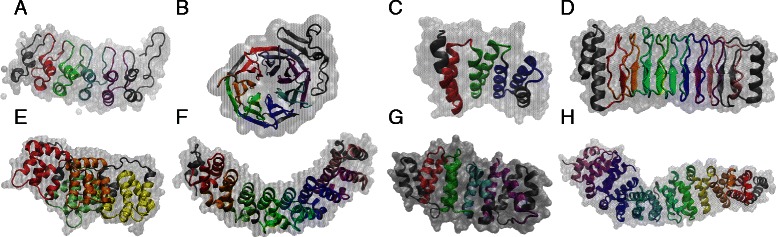
Repeat proteins are formed with tandem arrays of repeats. The crystal structures of members of different repeat protein families are shown, with the backbone colored according to the repeated units. The molecular surface of the repeat array is drawn in transparent gray. **a** ANK family (PDB:1IKN, chain D), **b** WD40 family (PDB:1ERJ, chain A), **c** TPR family (PDB:4GCO), **d** LRR family (PDB:4NKH, chain A), **e** ANEX family (PDB:2ZOC, chain A), **f** PUF family (PDB:2YJY, chain A), **g** HEAT family (PDB:4G3A, chain A), and **h** ARM family (PDB:2BCT)

Typical repeat protein domains are made up of tandem arrays of ∼20-40 similar amino acid stretches that fold into elongated architectures of stacked repeating structural motifs, although unique “domains” are not trivial to define by structural inspection [6] (Fig. 1). Successful design of repeat proteins with novel functions based on simple sequence statistics [9] suggests that folding and functional signals can be partially segregated. Energy landscape theory predicts that foldable polypeptides are much easier to realize in the presence of symmetry as compared to asymmetric arrangements [10]. Funneled energy landscapes imply that patterns can form in different parts of the molecule with relative independence and subsequently assemble to higher order structures. This greatly reduces the folding search problem by efficiently arranging relatively small fundamental building blocks, or “foldons” in a repetitive fashion [11, 12]. Thus, due to the approximate translational symmetry, repeat proteins constitute excellent systems in which to study the coupling between sequential, structural and functional patterns.

The maximum entropy principle proposes a scheme for approaching the problem of extracting essential pair couplings from multiple sequence alignments of families of homologous proteins [13–18]. The main technical limitations confounding residue correlations are the transitivity of the correlations, the statistical noise due to the relative small number of available observables, and the phylogenetic dependence of the set of sequences assembled into a protein family [19]. Indirect interactions may generate the dominant correlations, and disentangling direct from indirect links is a fundamental step towards inferring the energetics underlying the observed couplings [14]. The application of direct coupling analysis (DCA) provides an efficient way of extracting meaningful information from the apparent junk of massive genomic data [20]. The mean structure of several protein domains can be reasonably well predicted from the statistical analysis of variations in large sets of sequences [18, 21, 22]. Strong deviations of the statistically coupled positions from the known domain structures lead to the exploration of the dynamical aspects of proteins that are related to biological function [23]. Likewise, specific interactions between domains can be characterized and good approximations to the interaction energetics can be obtained [15, 24–26]).

Other methods for inferring contact patterns from proteins sequences have been recently developed such as pseudolikelihood maximization (plmDCA [27]) and Gremlin [28], PconsC2, a deep learning approach to identify protein-like contact patterns [29], or combined implementations of various algorithms within a neural network such as metapsicov [30, 31]. These implementations were developed and tested mostly with globular protein domains, with varying degree of success.

In this work we show the limitations of the DCA methods developed for globular proteins when applied to repeat proteins. The translational symmetry of repeat proteins confounds the two point correlation introducing a strong bias at precisely the length scale of the repeated unit. We propose and implement an analogous procedure for quasi-translationally symmetric repeat proteins. The resulting correlation matrices allow for the identification local native contacts. Furthermore we propose a systematized way of selecting the main correlated pairs of positions from these matrices and set a minimum number of sequences needed to robustly use these procedures. These implementations can be extended and included in the calculations on globular protein domains. Additionally, the correction we suggest for repeat proteins is general enough such that it can be applied to the most recent implementations, such as the ones mentioned above.

We apply the overall procedure to infer native contacts in more than 25 families of repeat proteins of the solenoid class III (Additional file 1: Table S1, some shown in Fig. 1) and found that some families have strong coevolutionary interactions mainly between repeats, while others mainly within single repeats. These observations may be linked to the functional characteristics of the families.

## Results and discussion

### Direct coupling analysis of repeat proteins

We obtained sequences of single repeated units for the families listed in Additional file 1: Table S1 from the PFAM database, version 27.0 [32]. Since a repeat domain is typically formed with multiple tandem copies of repeated units [6], the minimal sequence that includes an interface between repeats is composed of two consecutive units. We thus constructed multiple sequence alignments (MSA) of pairs of consecutive repeats for each family. The sets of sequences were corrected for phylogenetic bias and finite-size sampling as described in the Methods.

**Fig. 2:**
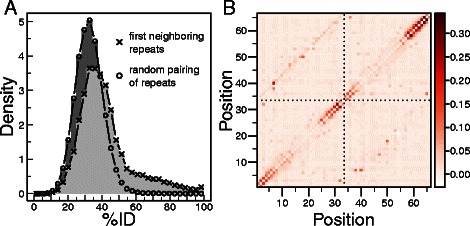
The sequence identity between repeated units can bias the inference of evolutionary couplings. Repeat sequences of the ANK familywere concatenated in a MSA of size 2*L*_0_=66 positions and ≈73000 sequences and co-variations were measured with direct information metric.**a** Sequence identity distributions between consecutive ANK repeats found in (x) natural proteins and (o) randomized pairs of repeats. **b** Direct information matrices between positions obtained without correcting (DI, upper half) or with proper equalization for repeat identity (DI _*id*_, lower half)

Direct information (DI) uses covariance in homologous protein sequences to deduce structural constraints. DI (eq 1) uncouples direct interactions from interactions mediated by a third residue on the complete sequence of the protein. The upper triangle of Fig. 2b shows the DI matrices for one of the most abundant repeat proteins, the ANK family. The typical length of these repeats is 33 residues, so values on columns/rows 1 to 33 and 34 to 66 correspond to interactions between residues within a repeat, while values on columns 1 to 33 and rows 34 to 66 correspond to interactions between residues on consecutive repeats. The values corresponding to pairs of positions on consecutive repeats reach comparable values to those within each repeated unit. There appears to be as much evolutionary correlations between residues on the same repeat as between residues in consecutive repeats. A question that arises is whether the strong signal between repeats is due to the inevitable similarity of the sequences of the repeat regions or to true coevolutionary interactions between neighboring repeats.

A close inspection of the couplings detected between repeated units reveals that the strongest signals are attributed to pairs of positions that are 33 residues apart (Fig. 2b, upper triangle). Since the ANK repeats aligned are of this precise length *L*_0_, these apparent interactions occur between residues that occupy equivalent positions in each repeat, i.e: the pair of positions (*i*,*i*+*L*_0_) corresponds to the *i*th residue on the first repeat and the *i*th residue on the second repeat. If repeats in proteins were identical, the interactions between residue *i* and *i*+*L*_0_ should get maximum DI values as these would show perfect co-variation. At the same time, the submatrix of positions between repeats should be identical to the submatrix of pairs of positions within the repeats. Thus, the identity between repeated units should be taken into account when evaluating correlations between repeats. One could be tempted to simply disregard the results for the *i*,*i*+*L*_0_ positions, arguing that these are caused by the repetitive nature of the system. Nevertheless, these pairs of positions may or may not correspond to actual contacts, as it will be shown below.

To characterize how the identity between neighbouring repeats affects the covariation analysis, we compared the distribution of the percentage of identical residues, *%* *I* *d*, between pairs of consecutive repeats, and between randomly assembled pairs of repeats (Fig. 2a). For the ANK family, the distribution of *%* *I* *d* for random pairs is centered around 30 %, while the natural pairs show higher mean and a large tail towards higher *%* *I* *d* values (Fig. 2a). This higher similarity between pairs of consecutive repeats is expected to induce correlations between *i* and *i*+*L*_0_ positions, as observed. To compensate for the higher *%* *I* *d* between natural repeats we developed a correction factor that equalizes the effects of quasi-translational symmetry. This correction consists of calibrating the weight of each sequence in the natural neighbours according to the *%* *I* *d* between the component pair of repeats, and rescaling it so that it matches the expected frequency of *%* *I* *d* between random pairs of repeats of the same family (see Methods). We refer to the obtained values as DI _*id*_. Figure 2b shows the DI matrix corrected only for phylogeny and finite counts (upper triangle), together with the matrix that includes this additional factor DI _*id*_ (lower triangles). The strong symmetric (*i*,*i*+33) off-diagonal signal is attenuated, as expected if the signal originates from biases in the *%* *I* *d* distributions. Importantly, the DI values obtained for interactions between all other positions are not significantly affected by the *%* *I* *d* equalization (Additional file 1: Figure S1).

The same analysis was performed for all the other repeat protein families (see Additional file 1: Figure S2). Equivalent results are obtained for several families: for example TPR, CW and PENTAPEPTIDE families show a strong bias in the symmetric (*i*,*i*+*L*_0_) interactions. These also show a higher sequence identity between true first neighbouring repeats, which biases the inter-repeat couplings. There are some families, for example ARM, ANEX and PUF, which do not show a high (*i*,*i*+*L*_0_) signal on the DI matrices. For these, the distributions of *%* *I* *d* between true and random neighbours are similar, consistent with the notion that the symmetric signal is caused just by the bias in similarity between neighbouring repeats. Applying the *%* *I* *d* equalization to these families does not significantly change the DI values, showing that the correction is not detrimental to the overall procedure. There are some particular cases like the HEAT family which has a very rugged *%* *I* *d* distribution. We believe that these effects are caused by an insufficient number of effective sequences on the alignments, which cannot ensure a robust calculation of DI (*vide infra*). The HEXAPEP family has a strong signal on a diagonal (i,i+ *L*_0_/2), suggesting that PFAM definition of repeat may involve in fact pairs of repeats, as we confirmed contrasting with an available structure (Additional file 1: Figure S2). We also analyzed the PPR proteins, a family for which there are no associated structures in PFAM. Both DI and DI _*id*_ maps show a reasonably structured distribution of values which can be a good prediction of a contact map. There are no significant differences between DI and DI _*id*_ values as the identity of consecutive repeats is low, i.e. similar to the distribution of identities of random pairs of PPR repeats. We conclude that sequences of proteins that show quasi-translational symmetry should be treated with an additional correction factor to account for the biases that the internal sequence identity can bring about.

### Prediction of native contacts for repeat proteins

**Fig. 3:**
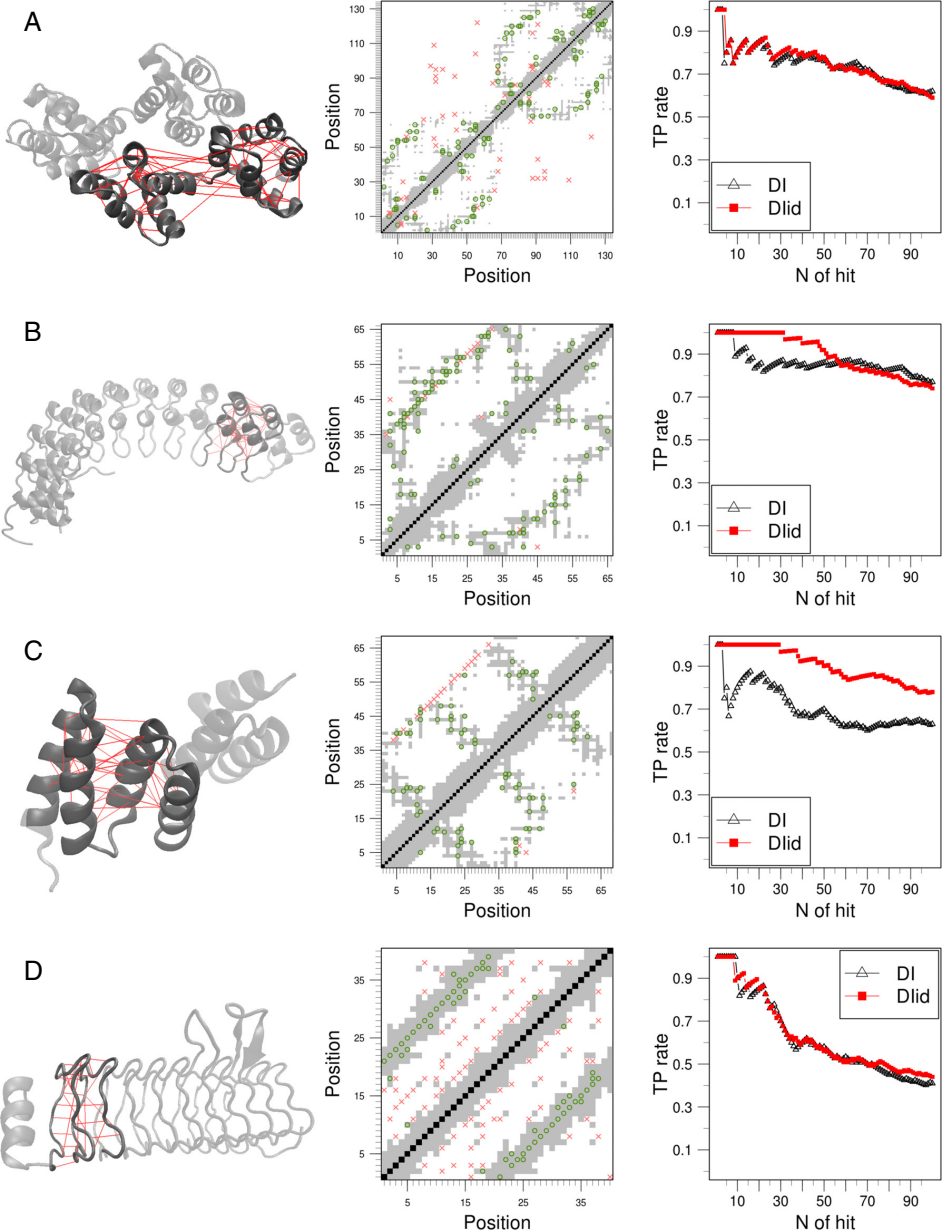
Native contacts can be predicted from the identity-equalized direct information DI _*id*_. On the center we show on grey shadow the contact map (closest atoms at distance lower than 8 Å) of representative family members **a** ANEX (PDB:2ZOC, chain A) **b** ANK (PDB:1N11, chain A) **c** TPR (PDB:4GCO). **d** PENTAPEPTIDE (PDB: 3DU1, chain X). On the upper triangle DI hits are marked in red crosses when they do not match a contact and on green circles when they do. On the lower triangle DI _*id*_ hits are marked in red crosses when they do not match a contact and on green circles when they do. On their side we show the structure used with the backbones as gray ribbons, and the first 20 predicted contacts along multiple repeat pairs in red. On the right we compare the true positive rate obtained using DI (black triangles) and DI _*id*_ (red squares) as predictor of contacts on the selected structure

Several high resolution structures for repeat proteins are available. These typically fold into elongated architectures where most members of a family display an overall similar topology (Fig. 1). We chose a representative structure for each family and mapped the numbering to the sequences of the MSA. On the other hand, we selected the main hits of DI and DI _*id*_ using the clustering method described in the Methods section. Figure 3 shows a comparison of the contact maps versus DI (upper triangle) and DI _*id*_ (lower triangle) hits. Green circles mark the coincidences and red crosses the DI or DI _*id*_ hits that are not contact in the reference structure. The pattern of evolutionary interactions inferred from the clustering of DI _*id*_ is remarkably similar to the experimental contact map for most families (Fig. 3 and (Additional file 1: Figure S3). The signals from the pairs of positions of consecutive repeats (*i*,*i*+*L*_0_) do not always correspond to a high contact probability, yet if present they are confidently detected.

One of the longest pairs of repeated units we study belongs to the ANEX family (2*L*_0_≈ 132 residues). The DI _*id*_ hits strongly resemble the average contact map, with 56 out of the 76 DI _*id*_ pairs found (∼73 *%*) within contact distance (Fig. 3a). Even though DI _*id*_ matrix does not differ much from DI matrix, as expected because the *%* *I* *d* histogram of consecutive repeats does not differ much from the one of random repeats (Additional file 1: Figure S2), we detect a slight improvement. The true positive (TP) rate of both quantities according to the number of hits taken can be seen on the right panels of Fig. 3. Most of the pairs with high DI _*id*_ correspond to interactions within each repeat, with few interactions at the repeat interfaces, unlike the correlations found in other repeat proteins, such as the ANK family (Fig. 3b). For ANK family, the clustering procedure assigns 44 hits for DI _*id*_, 42 of which are found within contact distance. Most of these are found outside the usual binding site of these proteins – the *β*-hairpin motif [9]. For comparison, DI assigns 79 hits from which 62 are contacts.

Within the top 43 DI _*id*_ identified for the TPR family, 40 are typically found within contact distance in the experimental structures and most of the outliers are in regions physically compatible with the known structures (Fig. 3c). In this case, the DI _*id*_ highly impoves the contact prediction respect to DI.

A particular case is represented in the analysis of PENTAPEPTIDE family, which has contacts between residues *i* and *i*+*L*_0_ (Fig. 3d). In this case the equalization DI _*id*_ does not significantly reduce the detection of these pairs of positions as pairs with high correlation.

In Additional file 1 the results obtained for all families are shown. For example the ARM family, where only 12 interactions correspond to contacts among the 31 predicted (Additional file 1: Figure S3). In the case of the LRR family, few interactions appear as outliers in DI _*id*_ distribution, and most of them have been observed to form close contacts between repeated units (Additional file 1: Figure S3). Few co-evolutionary interactions are assigned in the HEAT family, probably due to the limited number of available sequences (see below). Since there are no experimental structures associated to the NEB family, we cannot evaluate if the identified DI _*id*_ hits correspond with native contacts. This constitutes a prediction of the native contacts topology for this family.

### Distant couplings along a repeat array

Folding of repeat domains usually involves the cooperative formation of structures at a length scale that exceeds first neighbours [33]. Folding in some regions nucleates the folding of contiguous segments, allowing for a quasi-one-dimensional treatment of the dynamics [34]. A natural question that arises is how do evolutionary couplings in and between repeats change as the separation between the repeats increases.

**Fig. 4:**
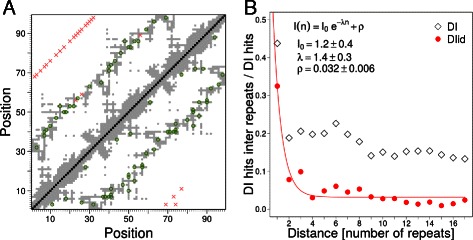
Correlations along ANK repeat arrays. **a** Direct information first 50 hits over a contact map (PDB:1N11,A, resid 436 to 534) calculated for three consecutive ANK repeats without (upper triangle) or with (lower triangle) the DI _*id*_ equalization. **b** Proportion of DI (black diamonds) and DI _*id*_ (red circles) hits between repeated units for alignments of *n*-th neighbours. The red line is a non-linear fit of the DI _*id*_ data to an exponential decay

An analogous correction to the weights of the sequences must be made to treat *n*-neighbours interactions (lower triangle of Additional file 1: Figure S5 for the uncorrected DCA of three consecutive repeats of the ankyrin family). When the proper equalization is performed, the symmetric signals attenuate and the true coevolutionary correlations appear (DI _*id*_ lower triangle of Additional file 1: Figure S5). In principle the correction to the symmetric (*i*,*i*+*n* *L*_0_) interactions can be applied to arbitrarily large repeat proteins. Yet the sampling needed is much larger and the computing time growths as L ^2^, restricting the application to longer repeat arrays. Since in ANKs, as in most of the repeat protein families, interactions are concentrated at relatively short sequences separations, we reconstructed a DI _*id*_ matrix from a parallel calculation of repeat pairs. For first neighbours we estimated DI _*id*_ as described previously, and for second neighbours we concatenated the sequences in an MSA of size 2*L*_0_. The reconstructed matrix for all interactions is very similar to the one calculated on the whole three-repeat MSA (Additional file 1: Figure S5), facilitating the application of the analysis for larger repeat arrays. On Fig. 4a we show the first 50 hits of DI (upper triangle) and DI _*id*_ (lower triangle) overlaid on a contact map of three consecutive ANK repeats (PDB:1N11,A; resid 436 to 534). The necessity of the equalization becomes more evident when longer repeat arrays are considered.

We observed that as the separation between repeats increases, the DI and DI _*id*_ between repeats decay significantly (Fig. 4b). True repeat pair interactions are less frequent, and this is reflected in the evolutionary couplings between units. While DI _*id*_ decays to almost zero, there remains a fraction of DI hits between distant repeats, indicating the need for the equalization for similarity along the repeat array. The number of interactions between repeats decreases roughly exponentially with repeat separation, with a half-length of about 1.4 repeats (Fig. 4b), suggesting that the evolutionary interaction length of Ankyrin repeat arrays is ∼1.5 units.

### Robustness and confidence of the analysis

For a robust calculation of the DI one must have a sufficiently large number of effective sequences to approximate the marginal and joint probability distributions from the observed frequencies of occurrences of amino acids. Since there is no general principle indicating how many sequences are necessary and sufficient for robust estimation, we empirically quantified the minimum number of effective sequences in various repeat protein families.

We constructed subsets of alignments by recurrently removing random groups of sequences from each dataset of repeat pairs, and calculated DCA on each of these subsets. The reduction in the number of sequences typically decrease the absolute values of the high ranking DI _*id*_ matrix elements and at the same time increases the background DI _*id*_ signals (Additional file 1: Figure S6), making DI _*id*_ signals indistinguishable from the background for small sample sizes.

**Fig. 5:**
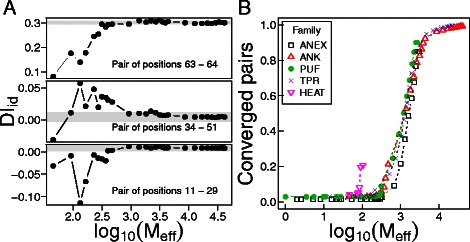
Robustness of the DI _*id*_ procedure. Subsets of alignments were constructed by recurrently removing random groups of sequences from each dataset of repeat pairs. M _*eff*_ is the number of effective sequences used in the alignment. **a** Particular examples of the stability of DI _*id*_ assignments as sampling changes on the ANK family. The gray shadow delimits the 1 % fluctuation interval set as a convergence criteria. **b** Overall stability of the DI _*id*_ assignments in several repeat protein families

For well determined parameters we expect the true value will be better estimated as sampling increases. Examples of the robustness of the DI _*id*_ assignments are shown in the panels of Fig. 5a. While the DI _*id*_ of some residue pairs can be confidently established with about 500 effective sequences, other pairs do not reach stable values even when all the available sequences are taken into account (Fig. 5a). To globally quantify the convergence of the DI _*id*_ matrix we evaluated how many of the residue pairs reach a limiting value within 1 % of the one obtained with the largest sample size. For every subset of sequences, *s*, we require that $|DI_{\textit {ij}}^{s}-DI_{\textit {ij}}|<0.01 \cdot \left (\max (DI)-\min (DI)\right)$, where $DI_{\textit {ij}}^{s}$ is the DI between position *i* and *j* calculated over the *s*-th subset, *D* *I*_*ij*_ is the DI on the largest set of sequences, and max(*D* *I*) and min(*D* *I*) are the maximum and minimum values for all positions in all subsets. Additionally all subsets larger than the subset *s* one must have a standard deviation lower than 1 % of the standard deviation of the DI values from all the subsets. If a residue pair fulfills these conditions, we say it has converged at the particular *s* sample size. We quantified how many of the residue pairs satisfy the convergence criteria at various sample sizes (Fig. 5b). The best sampled families, ANK and TPR, contain enough sequences to converge the DI _*id*_ for almost all residue pairs of consecutive repeats. Reducing the number of input sequences results in a loss of convergence of some sites; the DI _*id*_ of around 90 % of the residue pairs can be confidently established with about 10 % of the total sequences (*M*_*eff*_≈3000)(Fig. 5b). If the subsamples are further reduced, the proportion of positions that converge drops catastrophically. Yet even more relaxed criteria for convergence give confident results for the high-ranking DI pairs, as exemplified by the PUF and ANEX families (Fig. 5b). However the samples for the HEAT family are not sufficient to confidently quantify repeat pairs co-evolving.

## Conclusions

Repeat proteins are formed with various tandem repetitions of similar amino acid stretches. Due to the approximate translational symmetry, regions in proximity in the amino acid chain show similarities in their sequence patterns, which can result in close to perfect co-variation in a multiple sequence alignment and hence bias the inferred interactions between residues (Fig. 2). To compensate for this natural bias we developed an equalization that re-weights each sequence in the multiple alignment to account for correlations characteristic of the protein family. This procedure reveals the true co-evolutionary signals in the case of strong biases, importantly leaving the quantifications unchanged in the absence of bias. One cannot simply disregard the results for the *i*,*i*+*L*_0_ positions, arguing that these are caused by the repetitive nature of the system, as these pairs of positions may or may not correspond to actual contacts in different families. For example, in the PENTAPEPTIDE family (Fig. 3d) all pairs *i*,*i*+*L*_0_ are true contacts thus the symmetric interactions cannot be ignored. Conversely, Fig. 3c shows the contact map of TPR pairs of repeats where most of these pairs of positions *i*,*i*+*L*_0_ are not in contact. Hence, it is necessary to apply a general method that can distinguish which of these pairs of positions can be safely predicted as true contacts.

In this work we tested this correction for the mean field DCA method, but the correction can be applied to other methods. As an example, we applied the correction to plmDCA (Additional file 1: Figure S8). We see that there is an improvement of the contact predictions, comparable to the application of the correction to mean field DCA. We are confident that the same correction can be included in other methods to avoid biases due to the repetitive nature of these proteins.

The DI _*id*_ metric resulting from this equalized DCA is a good predictor of native interactions at the sub-domain level for proteins with a quasi translational symmetry, similarly to the original DI metric for globular proteins [19]. The highest ranking DI _*id*_ pairs are usually found in spatial proximity in all of the repeat protein families analyzed (Figs. 3 and Additional file 1: Figure S3). Interestingly, the patterns of co-evolutionary interactions are not a random subset of all the native-interactions, but segregate into particular groups in each family. Some families display relative high inter-repeat correlations, while in others the repeats appear to be independent evolutionary units. In general, the families we study show the same amount of interactions in repeated units as between repeat-units (Additional file 1: Figure S7), which can be related to the coupling of the folding of the repeat-arrays.

In their native environment, most repeat proteins participate in binding other macromolecules, and are thus expected to show co-variations in the positions that correspond to the binding interfaces. We observed that some architectures do show higher co-variations at the typical binding interface, like the nucleic-acid binding PUF family, while in the ubiquitous ANK family the typical binding interface is depleted of DI _*id*_ pairs.

A reliable estimation of DI requires a sufficiently large number of sequences. This number depends on the length, the topology and the ontology of the proteins under scrutiny. We empirically quantified the minimum number of effective sequences needed by analyzing subsamples of repeat protein families (Fig. 5). In most families we found that ∼90 % of the residue pairs can be confidently established with ∼3000 sequences (Fig. 5). The highest ranking DI interactions confidently predict native contacts even for much scarcer sampling.

Repeat proteins usually fold cooperatively several consecutive repeats [33]. Nucleation of the folding in some region facilitates the folding of contiguous segments, allowing for a quasi-one-dimensional treatment of the dynamics [34]. We found that the statistical couplings calculated from sequence variations in the ANK family decay roughly exponentially (Fig. 4) as the separation between repeats increases. The predicted global correlation length of ∼1.4 repeated units is remarkably close to that inferred from statistical mechanical analysis of folding experiments [35, 36] and folding simulations [37]. These predictions are based on approximating long-range covariations from sets of pair-wise inter-repeat interactions, allowing for the application of the procedure for arbitrarily large structures for which an exact calculation would be computationally prohibitive.

## Methods

### Selection of repeat protein families

We detected 159 PFAM accession numbers repeated more than once in a same PDB chain among all PDB entries classified as repeat proteins in the database RepeatsDB [38]. We chose to analyze all the repetitive domains which appear in the structures catalogued in class III.

We used HMMER [39] to get all the PFAM assignments [40] that match these structures. We kept only those repeats whose length is less than 70 residues, that are repeated at least once in the same protein, and which have an associated structure in the PFAM database [40]. Families analyzed are listed in Additional file 1: Table S1.

### Multiple sequence alignments

We obtained the MSA (multiple sequence alignment) for repeat units with NCBI data from the PFAM [40] database. For each MSA we ignored the columns that contain gaps in more than the 70 % of the members. The remaining number of residues in each case is referred as *L*_0_, the typical lenght of a repeat-unit. In order to reconstruct tandem arrays of repeats, we concatenated the sequences that belong to the same protein according to identifier in the PFAM’s alignments, and for which the sequence separation is less than *L*_0_/3. We analysed MSAs which have a number of sequences larger than 1500. The alignment thus generated is referred as first neighbour alignment and has *L*=2*L*_0_ columns (positions) with *M* rows (sequences) for each of the prototypical families of repeat proteins listed in Additional file 1: Table S1.

To make three or larger repeats MSAs we followed an analogous procedure, imposing the listed restrictions to consecutive repeats.

### DCA calculations

On every constructed MSA we performed DCA using the matrix inversion method detailed in [18]. To correct for the phylogenetic bias in the ensembles of sequences, we weighted them with the Henikoff and Henikoff heuristic [41], by assigning a weight $ w_{i}=\sum _{j} \frac {1}{r_{j}\cdot s_{j}^ i}$ to each sequence. *r*_*j*_ is the number of different amino acids present in position *j* of the MSA and ${s_{j}^{i}}$ is the number of sequences that have the same amino acid on position *j* than sequence *i*. We approximated the effective number of sequences as $M_{\textit {eff}}=\sum _{i} w_{i}$. We calculated direct information (DI) as:

(1) $$ DI_{ij}=\sum\limits_{A,B}P^{dir}_{ij}(A,B)\ln\left(\frac{P^{dir}_{ij}(A,B)}{f_{i}(A)f_{j}(B)}\right)   $$

where *f*_*i*_(*A*) is the marginal frequency of amino acid *A* at position *i* of the MSA, *f*_*j*_(*B*) is the marginal frequency of amino acid B at position *j* of the MSA and $P^{dir}_{\textit {ij}}(A,B)$ is the probability of having amino acid *A* at position *i* and amino acid *B* at position *j* simultaneously generated by the direct coupling between these pairs of residues [18].

### DI _*id*_ calculation

To account for the self-similarity of repeated units, we weighted the sequences according to the sequence identity (*%* *I* *d*) of a pair of repeats. We calculated the frequency a sequence has a determined *%* *I* *d* between its repeats (*ν*(*%* *I* *d*)) and the probability of having the same *%* *I* *d* between pairs of repeats of the same family, but belonging to different proteins, *ν* ^*random*^(*%* *I* *d*). Since aligned repeats have L _0_ residues each, the *%* *I* *d* can only take discrete values *n*/*L*_0_ with *n* an integer between 0 and L _0_. We weighted each sequence by:

(2) $$ {w^{c}_{i}}= w_{i} \frac{\nu^{random}(\%Id=n/L_{0})}{ \nu(\%Id=n/L_{0})}  $$

where *w*_*i*_ is the Henikoff weight of a sequence that has *%* *I* *d*=*n*/*L*_0_. The DCA calculations that include these weights are referred to as DI _*id*_.

### Finite-size correction

The finite-size of the ensemble of sequences generates spurious correlations that must be corrected. By scrambling each of the columns of a natural MSA we generate MSA_*IM*_ which keeps the marginal frequencies of the amino acids in each position but breaks all true correlations. We calculated direct information for this site-independent alignment and subtracted the results from the direct information calculated on the original MSA. These values are presented in the matrices DI and DI _*id*_.

### Selection of top DI _*id*_

For several globular domains it has been shown that native contacts can be inferred from the inspection of the top-list of residue pairs according to the DI ranking [19]. There is no established way to discern the minimum value of DI to be used as the cutoff, as these depend on the topology of the fold, the sampling of sequences and the details of the method used to obtain DI, thus 50 to 200 pairs are empirically used [19, 24]. Since domains of repeat proteins are composed with multiple copies of repeated units, we asked whether DI and DI_*id*_ metrics are useful predictors of direct native interactions at the sub-domain level. We observed that the absolute values of DI we calculated for pairs of repeats are lower than those computed for globular domains, (Fig. 2 and Additional file 1: Figure S2), complicating the distinction of positive DI outliers from the background signal. We developed a clustering method to systematically delimit the true positive interactions. We first calculated the euclidean distance between each pair of DI values as $dDI_{a,b} = \sqrt {(DI_{a} - DI_{b})^{2}}$; and made a hierarchical clustering of the obtained distances. To delimit the clusters we used the dynamic tree cut method [42], which allows us to distinguish nested clusters. We found that most of the DI pairs fall in one big cluster which we assigned to the background signal (Additional file 1: Figure S4). The other clusters have fewer members and constitute outliers of the normal distribution. We consider the true coevolutionary signals as those within small clusters of positive DI values.

### Structural data

All structural data has been downloaded from the PDB database [32], and corresponding IDs are referred.

### Contact maps

Contact maps are a two dimensional representation of structural information. In these matrices, each position represents the interaction between two residues, scoring one when the residues are in contact and zero when they are not. We define that two residues are in contact when their closest heavy atoms are at less than 8 Å, following the definition of [18].

### Data accessibility

MSAs data from pfam.xfam.org, version 27.0 using PFAM Identifier [32]. Structure models from www.pdb.org, using PDB ID [43].

All calculations and analysis have been done with R scripts available at https://github.com/roespada/DCAforRpackage.git.

## Ethics

The authors declare that this study does not involve humans or animals.

## Additional file

Additional file 1 **Supplementary material file, including figures and tables mention in the main manuscript.**
