# Supplementary material for: Capturing coevolutionary signals inrepeat proteins
Source: BMC Bioinformatics. 2015 Jul 2;16:207. doi: 10.1186/s12859-015-0648-3 (PMC4489039; doi:10.1186/s12859-015-0648-3)
Supplement: Supplementary file 1 — Supplementary material file, including figures and tables mention in the main manuscript. [file 12859_2015_648_MOESM1_ESM.pdf]

Additional file 1
Capturing coevolutionary signals in repeat proteins

Rocío Espada[‡] R. Gonzalo Parra[‡] Thierry Mora[§] Aleksandra M. Walczak[⋆] Diego U. Ferreiro[‡*]

[‡] Protein Physiology Lab, Dep de Química Biológica, Facultad de Ciencias Exactas y Naturales, UBA-CONICET-IQUIBICEN, Buenos Aires, Argentina
[§] Laboratoire de physique statistique, CNRS, UPMC and École normale supérieure, 24 rue Lhomond, 75005 Paris, France
[⋆] Laboratoire de physique théorique, CNRS, UPMC and École normale supérieure, 24 rue Lhomond, 75005 Paris, France

[*] To whom correspondence should be addressed. Email: ferreiro@qb.fcen.uba.ar

DI and DI_{id}calculations over repeat protein families.

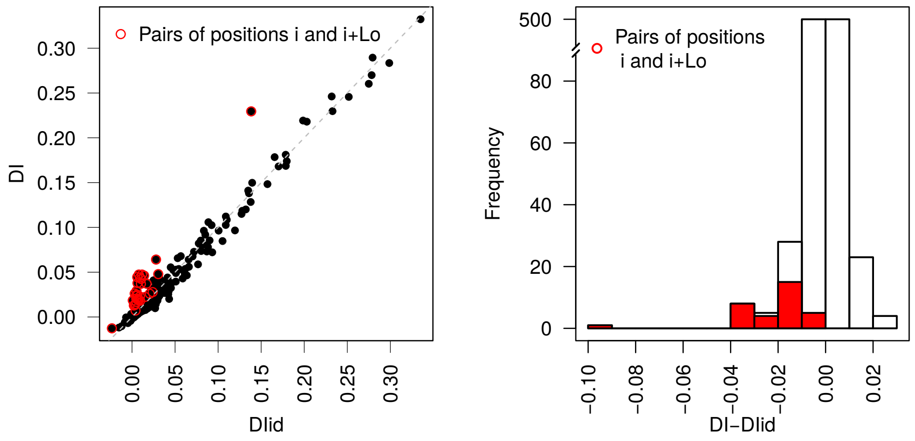

Figure S1: Comparison of DI and DI_{id} values. Left, DI values versus DI_{id} values for every pair of positions at the FNA. Pairs of positions representing the pairs between positions i and $i + L_o$ are coloured in red. It can be seen that most pairs do not modify their values when applying the equalization for sequence identity. Left, histogram of the difference DI-DI_{id}. Pairs of positions i and $i + L_o$ changed the most, while most values remains similar.

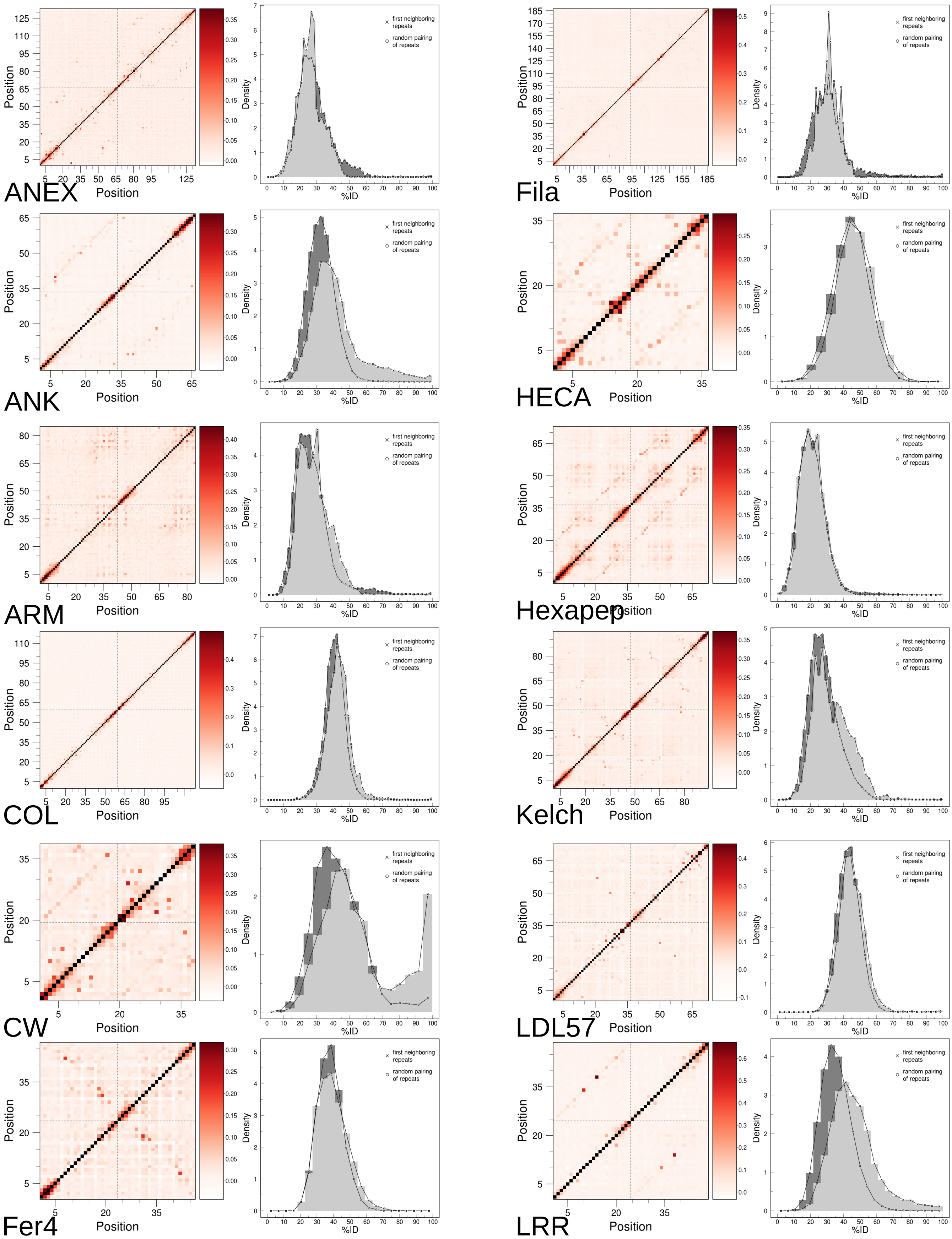
ANEX
Fila
ANK
HECA
ARM
Hexapep
COL
Kelch
CW
LDL57
Fer4
LRR
Position
Density
%ID
first neighboring repeats
random pairing of repeats

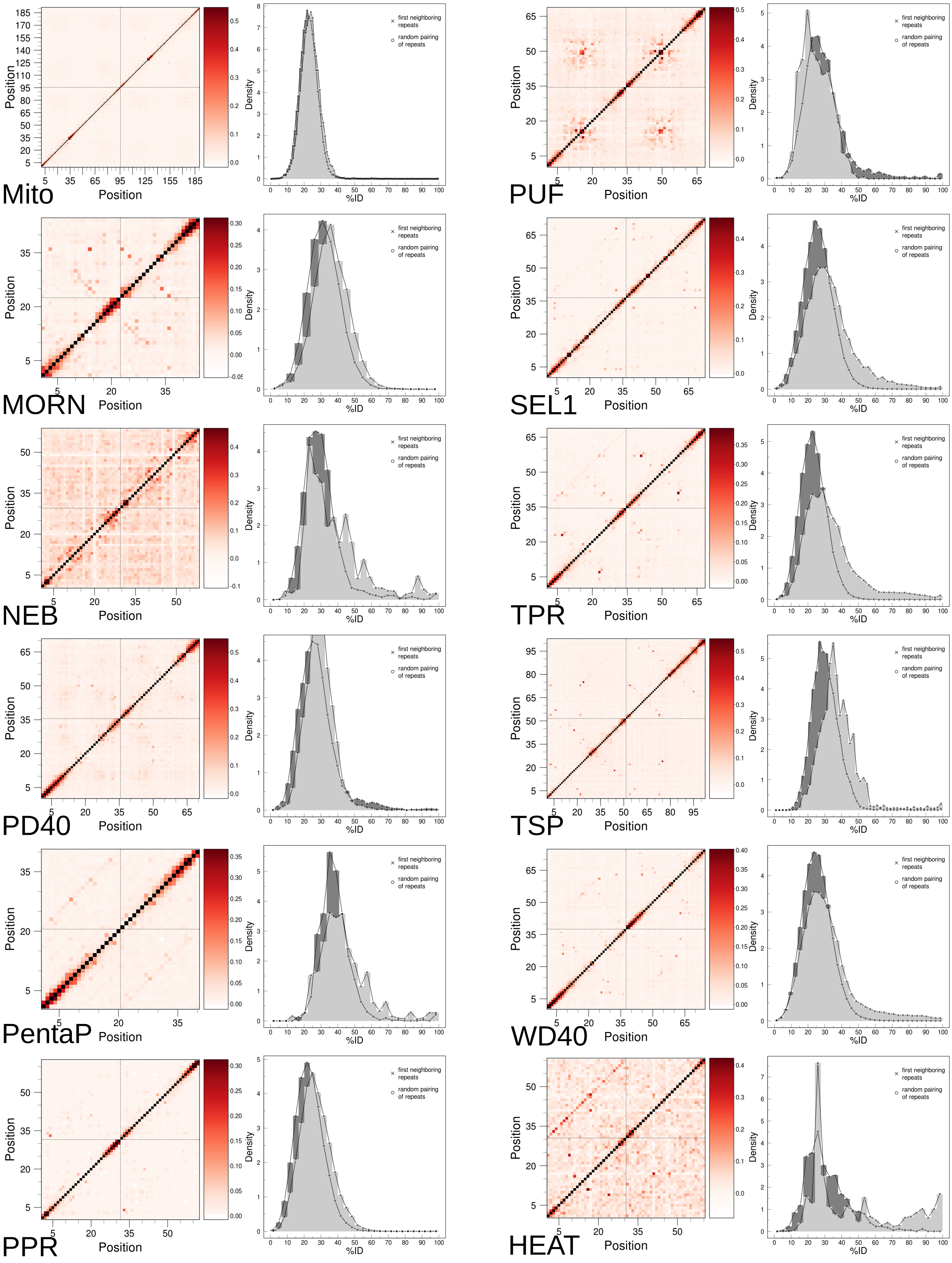

Figure S2: For each family, the red matrix is DI on the upper triangle and DI_{id} on the lower triangle; the second panel has the comparison between histograms of %Id for the FNA (first neighbours repeats - x) and the RPA (random pairs of repeats alignment - o).

DI and DI$_{id}$ for representative structures of repeat protein families.

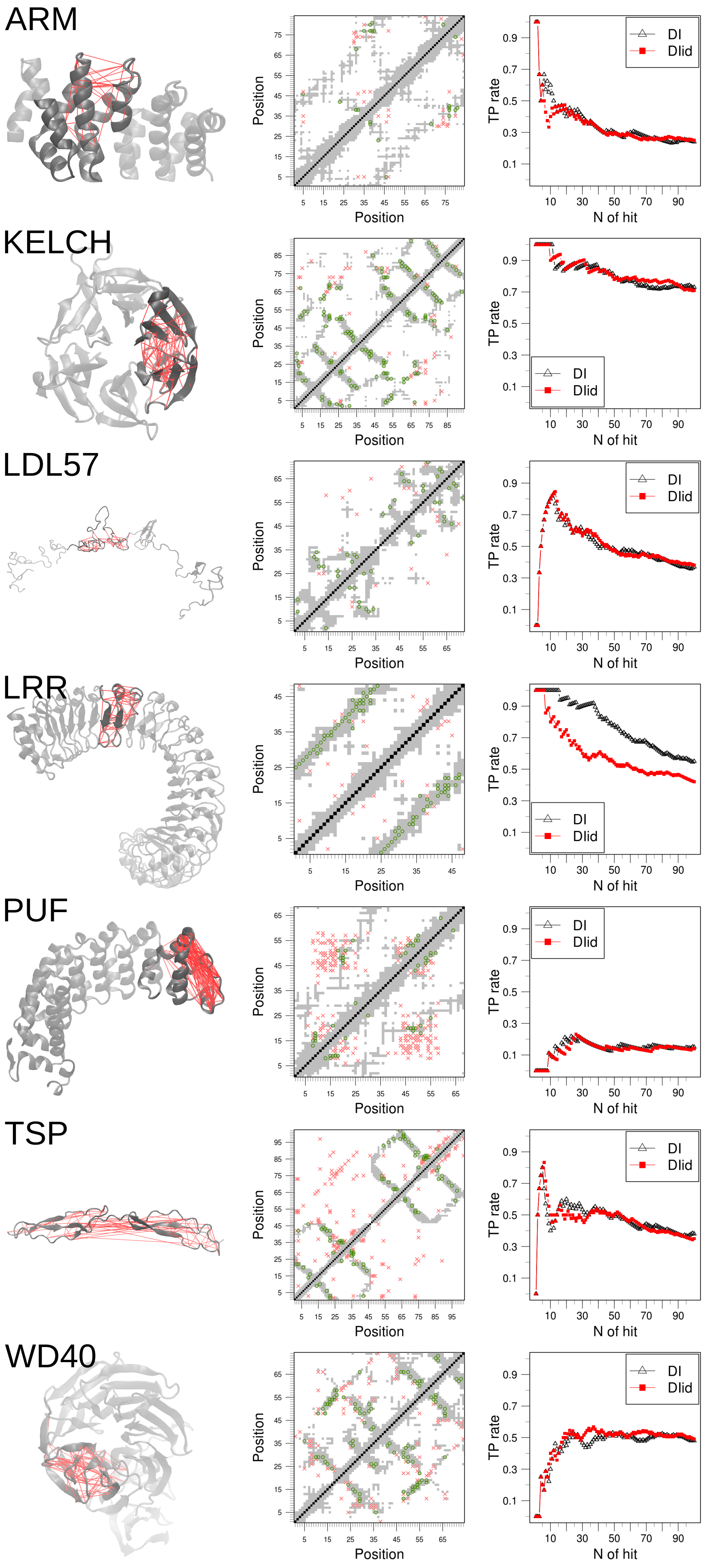

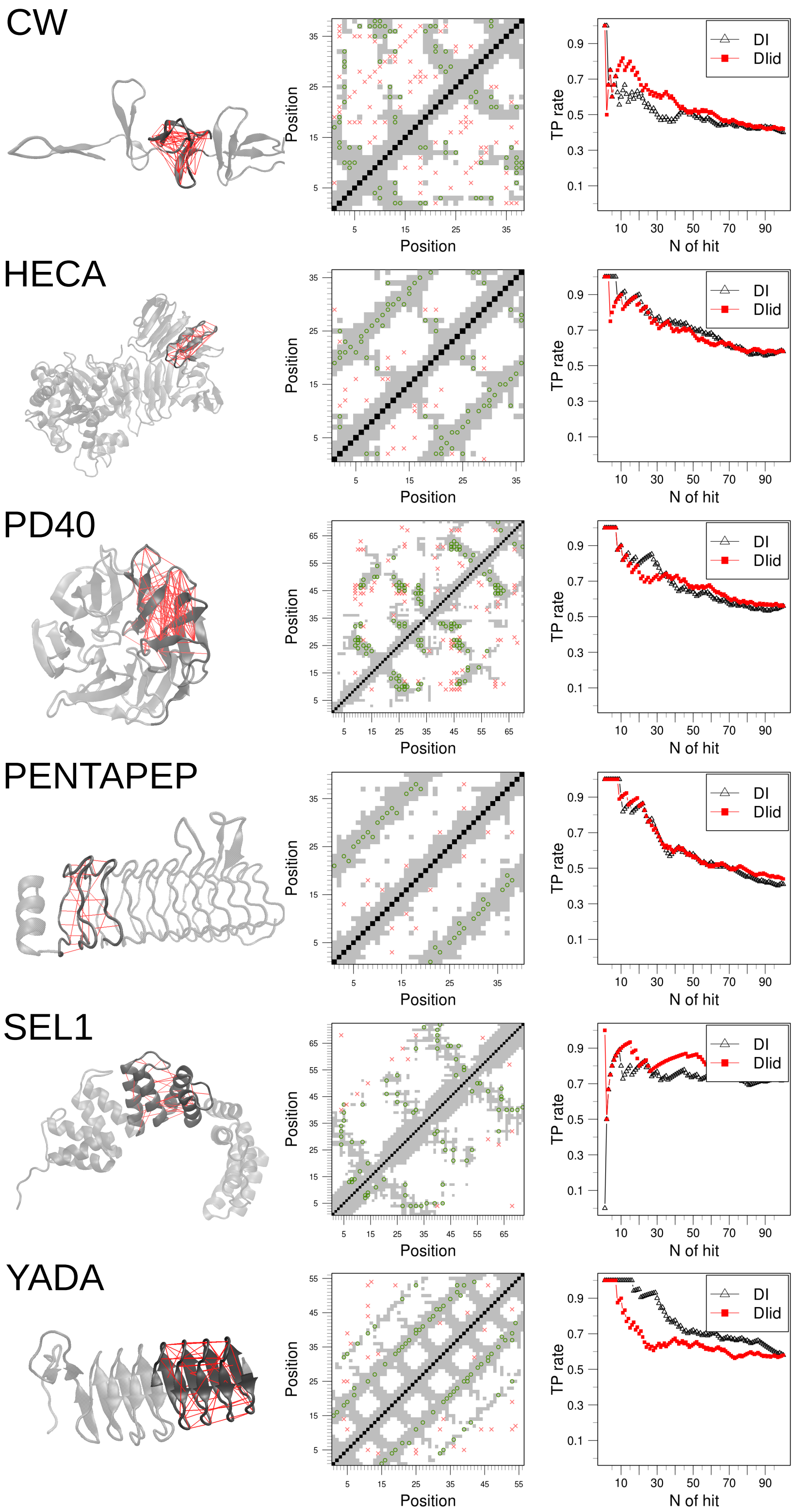

Figure S3: Analysis for different protein families. On the center we show on grey shadow the contact map (closest atoms at distance lower than 8 Å) of representative family members. On the upper triangle the DI hits are marked in red crosses when they do not match a contact and on green circles when they do. On the lower triangle DI_{id} hits are marked in red crosses when they do not match a contact and on green circles when they do. On their side we show the structure used with the backbones as gray ribbons, and the first 20 predicted contacts along multiple repeat pairs in red. On the right we compare the true positive rate obtained using DI (black triangles) and DI_{id} (red squares) as predictor of contacts on the selected structure.

Selection of top DI and DI_{id}.

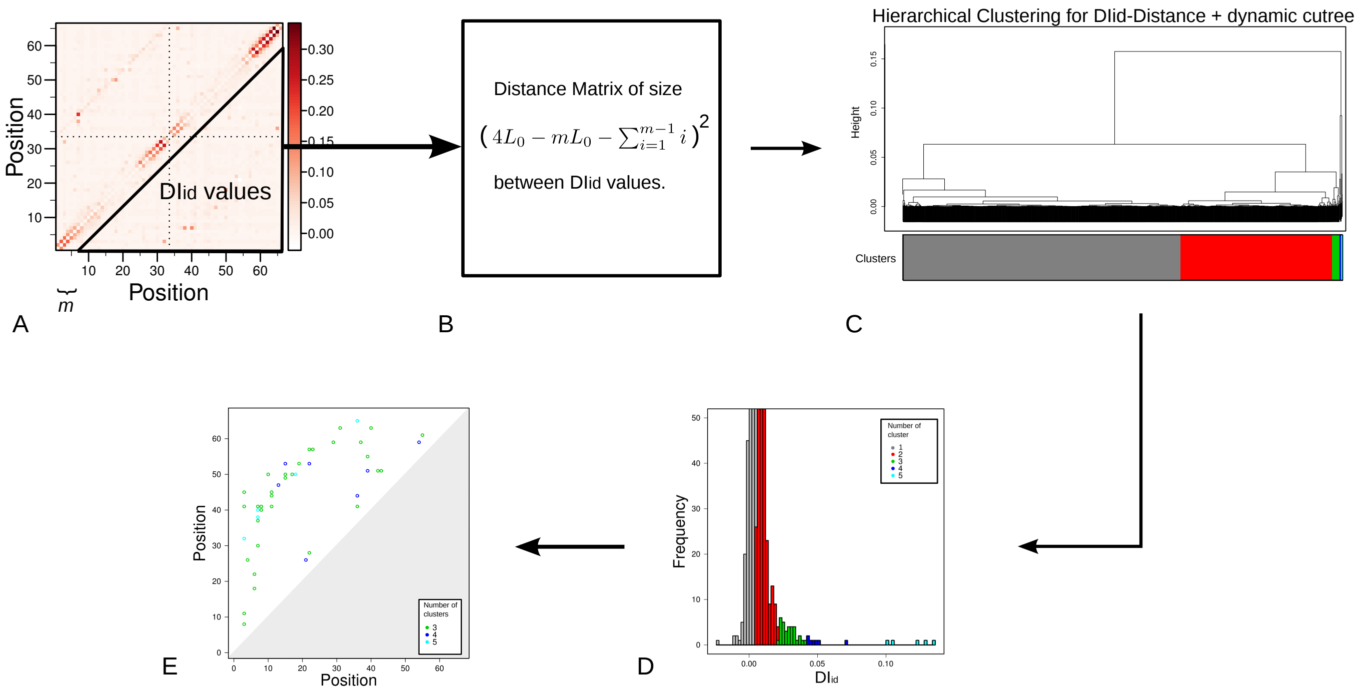

Figure S4: Schema of the process to select top DI and DI_{id} values. From the DI_{id} matrix (A) we take the values of DI_{id} of pairs of positions closer to m residues in sequence (we use $m = 5$). We calculate the euclidean distance between each pair of DI_{id} values and dispose them in a distance matrix (B). This matrix is used as input for performing a hierarchichal clustering represented in the dendogram at (C). Using the R package dynamic tree cut we group the DI_{id} values into clusters, as can be seen in colour code below the dendogram. In D we plot an histogram of the DIid differentiating according the cluster to which the DI_{id} value belongs. From this we choose entire clusters to be considered positive DI_{id} hits, which we plot again in the original matrix representation (E).

Distant couplings along a repeat-array

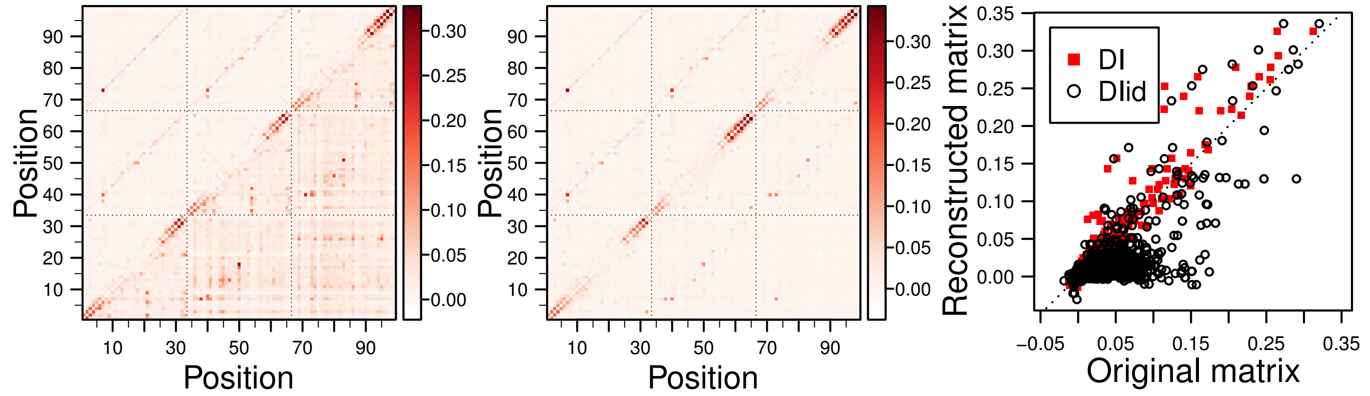

Figure S5: Left, upper triangle DI and bottom triangle DI_{id} for the three repeats alignment. Center, upper triangle DI and bottom triangle DI_{id} calculated from different alignments (first neighbours and second neighbours pairs) and reconstructing the matrix. Right, comparison of the DI and DI_{id} values obtained on the first two panels.

Robustness and confidence of the analysis

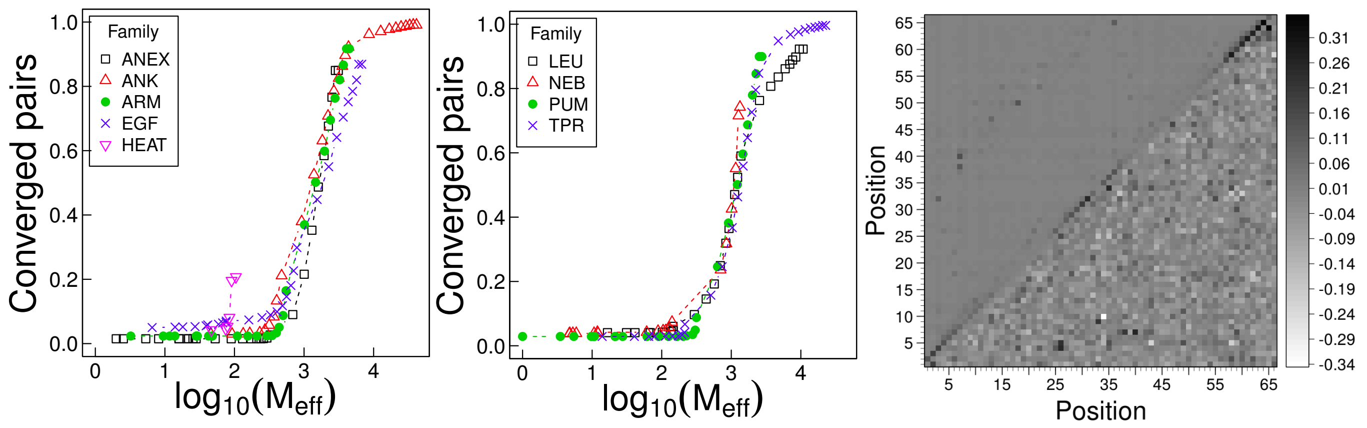

Figure S6: Left and center, for each family proportion of pairs of positions converged according to the criteria of the main text vs. the number of effective sequences on the alignment. Right, for the ANK family, example of DI_{id} matrix calculated over an alignment of around 70000 sequences (upper panel) and over an alignment of around 400 sequences (lower panel).

Coupling between and within repeats

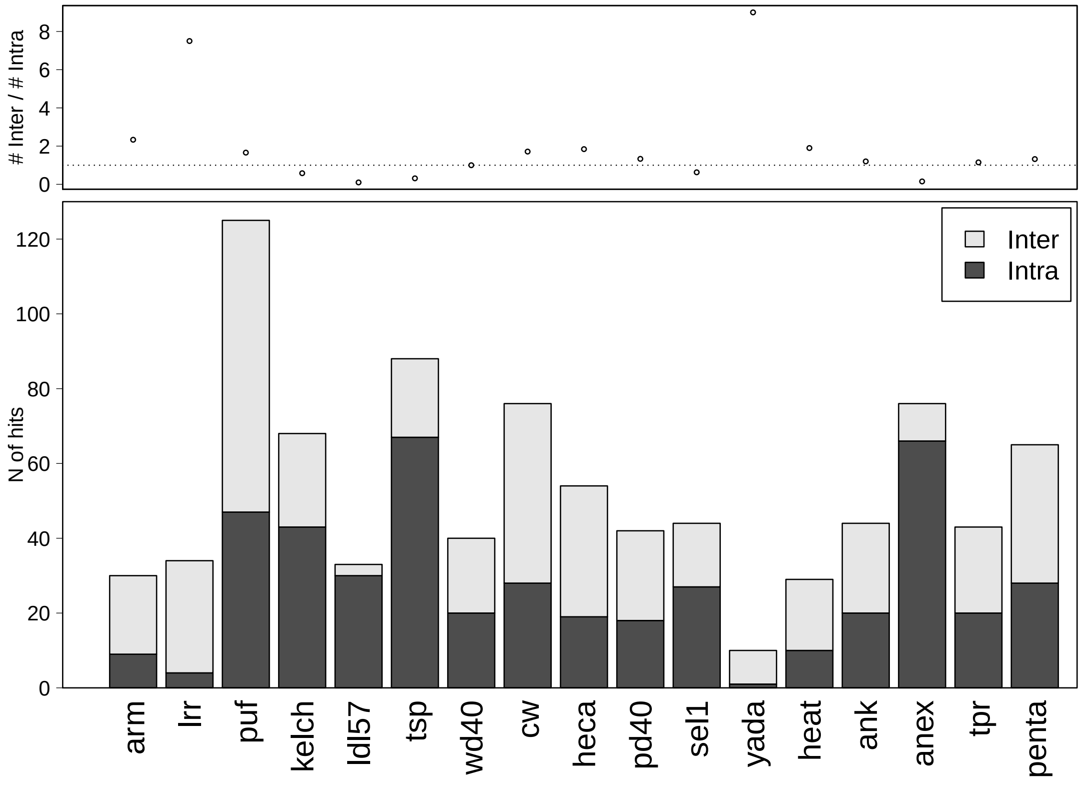

Figure S7: Analysis of number of DI_{id} hits in pairs of positions on the same repeat (intra) and in consecutive repeats (inter) for several repeat protein families. At the upper panel we show the ratio between the number of hits inter and the number of hits intra. If the ratio is shorter than 1 (is below the dot line) the family shows more coevolutionary signal in positions intra repeat than in positions inter repeats. In the panel below we show the number of DI_{id} hits coloured according to the inter/intra classification.

plmDCA and plmDCA$_{id}$ for representative structures of repeat protein families.

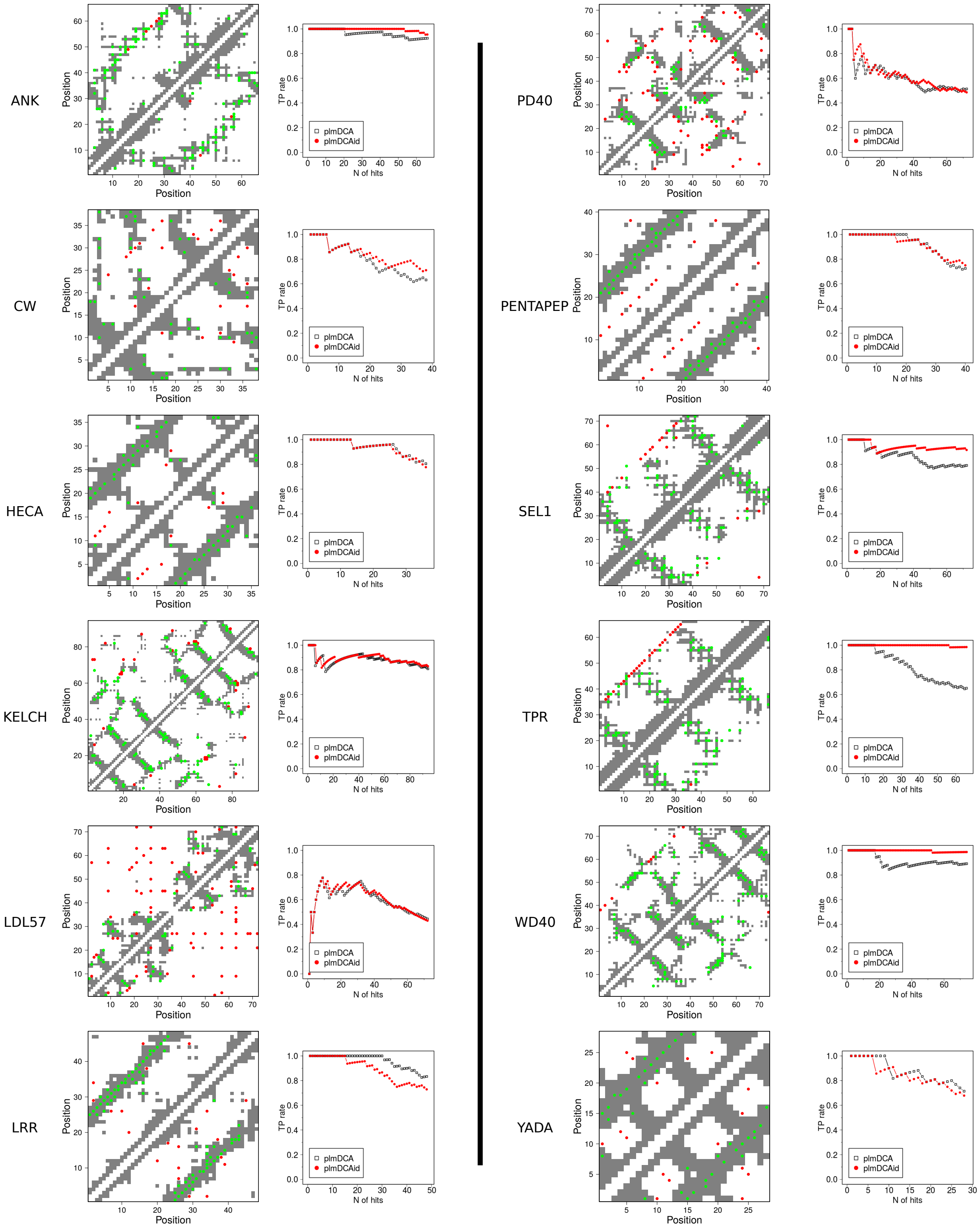

Figure S8: We applied the same correction to plmDCA. For each family we show in the right panel the contact map for a pair of repeats (grey shadow), on its upper triangle the first L_0 hits for plmDCA (in green when it matches a contact and in red when it does not) and on its lower triangle the first L_0 hits for plmDCAid, the corrected plmDCA (in the same colour code). In the right panel we show the true positive rate in black for plmDCA and in red for plmDCAid.

Table S1: Repeat protein families analyzed. L is the number of residues of the sequences on the FNA; M is the number of sequences on the FNA.

Family name	Abreviation	Pfam Identifier	$L = 2L_0$	M
ANKYRIN	ANK	PF00023	66	72908
ANNEXIN	ANEX	PF00191	132	4264
ARMADILLO	ARM	PF00514	84	6911
COLLAGEN HELIX	COLLAGEN	PF01391	118	8052
CW BINDING 1	CW	PF01473	38	11359
4FE-4S BINDING DOMAIN	FER4	PF00037	46	3780
FILAMIN/ABP280 REPEAT	FILAMIN	PF00630	186	5935
HEAT	HEAT	PF02985	60	513
HEMOLYSIN-TYPE CALCIUM-BINDING REPEAT	HEMOLYSINCABIND	PF00353	36	17200
BACTERIAL TRANSFERASE HEXAPEPTIDE	HEXAPEP	PF00132	72	11391
KELCH_1	KEL	PF01344	94	12202
LEUCINE RICH	LRR	PF00560	48	26493
LOW-DENSITY LIPOPROTEIN RECEPTOR DOMAIN CLASS A	LDL_RECEPT_A	PF00057	72	10727
LOW-DENSITY LIPOPROTEIN RECEPTOR REPEAT CLASS B	LDL_RECEPT_B	PF00058	84	4228
MITOCHONDRIAL CARRIER	MITO CARR	PF00153	190	21922
MEMBRANE OCCUPATION AND RECOGNITION NEXUS	MORN	PF02493	44	16761
WD40-LIKE BETA PROPELLER REPEAT	PD40	PF07676	70	3775
NEBULIN	NEB	PF00880	58	3925
PENTAPEPTIDE	PENTAPEPTIDE	PF00805	78	3430
PENTATRICOPEPTIDE	PPR	PF01535	62	50637
PUMILIO	PUF	PF00806	68	3995
SEL1	SEL1	PF08238	72	28502
TETRATRICOPEPTIDE	TPR	PF00515	68	38866
THROMBOSPONDIN	TSP	PF00090	102	6256
WD40 OR BETA-TRANSDUCIN REPEAT	WD40	PF00400	74	88617
HEAD DOMAIN OF TRIMERIC AUTOTRANSPORTER ADHESIN	YADA_HEAD	PF05658	56	1676
